# Mixed-methods economic evaluation of the implementation of tobacco treatment programs in National Cancer Institute-designated cancer centers

**DOI:** 10.1186/s43058-021-00144-7

**Published:** 2021-04-09

**Authors:** Ramzi G. Salloum, Heather D’Angelo, Ryan P. Theis, Betsy Rolland, Sarah Hohl, Danielle Pauk, Jennifer H. LeLaurin, Yasmin Asvat, Li-Shiun Chen, Andrew T. Day, Adam O. Goldstein, Brian Hitsman, Deborah Hudson, Andrea C. King, Cho Y. Lam, Katie Lenhoff, Arnold H. Levinson, Judith Prochaska, Fabrice Smieliauskas, Kathryn Taylor, Janet Thomas, Hilary Tindle, Elisa Tong, Justin S. White, W. Bruce Vogel, Graham W. Warren, Michael Fiore

**Affiliations:** 1 Department of Health Outcomes and Biomedical Informatics, University of Florida College of Medicine, 2004 Mowry Road, Gainesville, FL USA; 2 University of Wisconsin Carbone Cancer Center, Madison, WI USA; 3 University of Wisconsin Institute for Clinical and Translational Research, Madison, WI USA; 4 Rush University Medical Center and Rush Cancer Center, Chicago, IL USA; 5 Washington University Siteman Cancer Center, St Louis, MO USA; 6 University of Texas Southwestern Medical Center, Dallas, TX USA; 7 University of North Carolina Lineberger Cancer Center, Chapel Hill, NC USA; 8 Northwestern University Feinberg School of Medicine and Lurie Comprehensive Cancer Center of Northwestern University, Chicago, IL USA; 9 Indiana University Simon Cancer Center, Indianapolis, IN USA; 10 University of Chicago Medicine Comprehensive Cancer Center, Chicago, IL USA; 11 Huntsman Cancer Institute, University of Utah, Salt Lake City, UT USA; 12 Dartmouth-Hitchcock Norris Cotton Cancer Center, Lebanon, NH USA; 13 University of Colorado Comprehensive Cancer Center, Aurora, CO USA; 14 Stanford Cancer Institute, Stanford University, Stanford, CA USA; 15 Barbara Ann Karmanos Cancer Institute, Detroit, MI USA; 16 Georgetown University Lombardi Comprehensive Cancer Center, Washington, DC USA; 17 University of Minnesota Masonic Cancer Center, Minneapolis, MN USA; 18 Vanderbilt University Medical Center Vanderbilt-Ingram Cancer Center, Nashville, TN USA; 19 University of California Davis Comprehensive Cancer Center, Sacramento, CA USA; 20 Philip R. Lee Institute for Health Policy Studies, University of California, San Francisco, CA USA; 21 Medical University of South Carolina Hollings Cancer Center, Charleston, SC USA; 22 University of Wisconsin Center for Tobacco Research and Intervention, Madison, WI USA

**Keywords:** Implementation costs, Economic evaluation, Mixed methods, Smoking cessation, Tobacco treatment

## Abstract

**Background:**

The Cancer Center Cessation Initiative (C3I) was launched in 2017 as a part of the NCI Cancer Moonshot program to assist NCI-designated cancer centers in developing tobacco treatment programs for oncology patients. Participating centers have implemented varied evidence-based programs that fit their institutional resources and needs, offering a wide range of services including in-person and telephone-based counseling, point of care, interactive voice response systems, referral to the quitline, text- and web-based services, and medications.

**Methods:**

We used a mixed methods comparative case study design to evaluate system-level implementation costs across 15 C3I-funded cancer centers that reported for at least one 6-month period between July 2018 and June 2020. We analyzed operating costs by resource category (e.g., personnel, medications) concurrently with transcripts from semi-structured key-informant interviews conducted during site visits. Personnel salary costs were estimated using Bureau of Labor Statistics wage data adjusted for area and occupation, and non-wage benefits. Qualitative findings provided additional information on intangible resources and contextual factors related to implementation costs.

**Results:**

Median total monthly operating costs across funded centers were $11,045 (range: $5129–$20,751). The largest median operating cost category was personnel ($10,307; range: $4122–$19,794), with the highest personnel costs attributable to the provision of in-person program services. Monthly (non-zero) cost ranges for other categories were medications ($17–$573), materials ($6–$435), training ($96–$516), technology ($171–$2759), and equipment ($10–$620). Median cost-per-participant was $466 (range: $70–$2093) and cost-per-quit was $2688 (range: $330–$9628), with sites offering different combinations of program components, ranging from individually-delivered in-person counseling only to one program that offered all components. Site interviews provided context for understanding variations in program components and their cost implications.

**Conclusions:**

Among most centers that have progressed in tobacco treatment program implementation, cost-per-quit was modest relative to other prevention interventions. Although select centers have achieved similar average costs by offering program components of various levels of intensity, they have varied widely in program reach and effectiveness. Evaluating implementation costs of such programs alongside reach and effectiveness is necessary to provide decision makers in oncology settings with the important additional information needed to optimize resource allocation when establishing tobacco treatment programs.

## Background

More than one in six cancer survivors in the United States is a current tobacco user [1]. Continued tobacco use by cancer patients is associated with multiple adverse outcomes including increased overall and cancer-specific mortality, increased risk for second primary cancer, and strong associations with increased cancer treatment toxicity [2, 3]. The adverse effects of smoking can lead to substantial incremental cancer treatment costs, estimated annually at $10,678 per patient (equivalent to a total of $3.4 billion) for attributable failure of first-line cancer treatment in the US [4]. Smoking cessation can reduce many of these patient risks [5–15], but little consideration has been given to how evidence-based methods can be effectively integrated into standard oncology clinical practice, including the costs associated with tobacco treatment program implementation [16–19]. Even though tobacco treatment has been recommended as an essential component of cancer care for those who smoke [20–22], and most cancer organizations [23, 24] and the Surgeon General [25] advocate for providing cessation assistance to cancer patients, most oncology providers do not routinely assist patients to quit smoking [26–28].

To address this research-to-practice gap, the National Cancer Institute (NCI) established the Cancer Center Cessation Initiative (C3I) in 2017 under the Cancer Moonshot Program with the specific objective “to help cancer centers build and implement sustainable programs to routinely address tobacco cessation with cancer patients” [29]. Forty-two NCI-designated cancer centers received funding for 2 years over two funding cycles (2017–2019 for Cohort 1, 2018–2020 for Cohort 2). In 2020, an additional 10 centers received 1 year of funding (Cohort 3). A significant strength of the C3I is its recognition of the need to address multilevel (i.e., program-level, practice setting, and outer context) factors in the implementation of tobacco treatment programs, such as tobacco screening, integration into clinical workflow, use of information technology, and systematic documentation and outcome reporting.

The present study examined the costs of implementing tobacco treatment programs among the first two cohorts in the C3I during the NCI-funded implementation phase. The C3I provides a compelling infrastructure to study implementation costs for several reasons. First, the initiative requires significant investments from cancer centers to hire tobacco treatment specialists and adapt clinical workflows to integrate tobacco treatment as their clinical standard of care. As costs may be a barrier to implementation of tobacco treatment in cancer care broadly, economic evaluations are typically necessary before health systems adopt new initiatives. Further, understanding implementation and operation costs and continuing to pursue cost-effective strategies is key to sustainment of tobacco treatment at participating cancer centers. Therefore, the C3I presents a unique opportunity to investigate the role and implication of costs to tobacco treatment program implementation and sustainability. Whereas early implementation success of the initiative has been recently reported [30, 31], implementation costs in the C3I have not been examined.

Costs associated with health system program implementation and operation are infrequently collected but critical for decision makers to ensure program success and sustainment [32]. Demonstrating the economic value of tobacco treatment is important for its integration into routine cancer care processes. One such study from 2009 evaluated the cost-effectiveness of a smoking cessation program implemented at the time of surgery for lung cancer [33] and found the program to be cost-effective at both one and 5 years post-surgery. The cost per quality-adjusted life year (QALY) was $16,415 at 1 year and $2609 at 5 years. In 2005, the Childhood Cancer Survivors Study [34] tested a tobacco cessation intervention consisting of peer-delivered counseling for adult survivors of childhood cancer who smoke. The study concluded that the cost of delivering the intervention was approximately $300 per participant, and the incremental cost-effectiveness of the intervention compared with control was $5371 per additional quit.

A more recent economic evaluation of smoking cessation programs was conducted in Ontario’s regional cancer programs in 2018 using simulation modeling [35]. The study evaluated the cost-effectiveness of two smoking cessation approaches—the prior basic smoking cessation program consisting of screening for tobacco use, advice, and referral and a more intensive program that included cessation medication, counseling, and follow-up. Compared with the basic program, the intensive program was both more effective and more costly, with an incremental cost-effectiveness ratio of $3367 (Canadian dollars) per QALY gained and $5050 per life-year gained for males, and $2050 per QALY gained and $4100 per life-year gained for females. Given the increasing evidence supporting the health benefits of smoking cessation after a cancer diagnosis [25], economic evidence from cancer care extends the broad evidence base that tobacco treatment programs provide good value compared with other healthcare interventions [36, 37].

The objective of this study was to contribute to our limited understanding of the resources required to implement tobacco treatment programs in cancer care settings and the economic implications of various program designs by estimating the costs of tobacco treatment programs at cancer centers participating in the C3I. Whereas the C3I was implemented based on the promise of earlier search, economic evaluation of the initiative is an essential step to document how C3I builds upon previous efforts in tobacco treatment for cancer patients. In the economic evaluation of implementation efforts, the use of qualitative approaches to complement the quantitative cost data enables the characterization of the contexts and perspectives within which monetary values can be interpreted. This complementary, mixed methods approach is particularly salient in implementation research because the outcomes [38] and costs [39] are highly dependent on contextual factors. Accordingly, this study applied a mixed methods approach [40] to (1) conduct a comparative cost analysis of the various approaches to implementing tobacco treatment programs, and (2) comprehensively describe the economic perspective of C3I implementation by incorporating detailed context-specific information.

## Methods

### Overview

Of the 42 cancer centers from the first two cohorts of the C3I, 15 centers reported on their operating costs. This study used a mixed methods, comparative case study design, in which each participating site (*n*=15) is conceptualized as a case [41]. Comparing multiple cases leverages variations across sites and allows for the investigation of contextual factors influencing implementation outcomes, such as cost. Consistent with the objectives of case study research, this study relies on multiple methods of data collection, as the convergence of multiple types of evidence enhances the credibility of the analysis [42]. The study is guided by the Consolidated Health Economic Evaluation Reporting Standards (CHEERS) [43].

### Setting

The setting for this study consisted of NCI-designated cancer centers participating in the C3I program designed to enhance the routine delivery of evidence-based tobacco treatment services. Participating institutions are required to overcome patient, clinician, practice, and health system barriers to providing evidence-based tobacco treatment services to their oncology patients who smoke, to achieve institutional buy-in that treating tobacco use is a component of organizational “standard of care,” and to create mechanisms to sustain tobacco treatment services beyond the implementation funding period of the initiative. Several factors position the C3I as a unique setting to evaluate the implementation costs of behavioral interventions in cancer care including the number and diversity in size, structure, and geography of the funded NCI-designated cancer centers; the range of implementation strategies and tobacco treatment approaches utilized; and the available reporting on standardized patient outcomes and cost measures.

### Data collection procedures

Tobacco treatment program data were reported by each site to the C3I Coordinating Center for the purposes of program evaluation. The Coordinating Center, based at the University of Wisconsin-Madison Carbone Cancer Center, provides scientific and technical assistance to grantees in integrating evidence-based tobacco treatment services into clinical care [30] and was responsible for data collection across sites. The Coordinating Center has developed metrics to assess the tobacco treatment programs using standard measures for patient outcomes, including reach and effectiveness. Reporting of these metrics to the Coordinating Center was required of each participating site on a twice-annual basis using Qualtrics forms (Qualtrics, Provo, UT). Additionally, cost reporting has been encouraged for sites, following a similar reporting frequency. Cost reporting was first introduced for the July–December 2018 reporting period and we herein include all sites with operating cost data reported at least once during that interval through the January–June 2020 period. Sites were asked to report their program costs retrospectively using a Qualtrics form that was developed by the lead author in collaboration with the Coordinating Center. Two pilot sites provided feedback on the cost data collection form prior to its rollout to all sites. For qualitative data collection, site visit interviews were conducted by Coordinating Center staff with the director of the cancer center at each site, principal investigator(s) of the C3I project, program staff, clinical or administrative leaders, IT staff, and/or other administrators. The study was determined to be exempt from institutional review board (IRB) approval as there were no patient-level data used, and the study was categorized as program evaluation by the University of Wisconsin-Madison IRB.

### Tobacco treatment program components

On the twice-annual surveys, sites reported whether they implemented the following types of evidence-based tobacco treatment components: in-person (individual or group) counseling delivered by a tobacco treatment specialist, telephone-based counseling delivered by a tobacco treatment specialist from the program (i.e., internal, other than referral to the quitline), cessation counseling delivered at the point of care, track and triage services delivered by interactive voice response system (i.e., TelASK), referral to the quitline, smokefreeTXT text messaging service, web resources (e.g., smokefree.gov), and cessation medications offered as part of the tobacco treatment program.

### Outcome measures

Standardized metrics were used to report on reach and effectiveness. Program reach was defined as the proportion of current smokers who were offered any type of tobacco treatment program, among current smokers with a clinical visit in the 6-month reporting period. Engagement in a program was defined as participation in an individually or group-delivered counseling program, in-person or via phone, fax or e-referral to a quitline, a website, or a text/mobile program, counseling regarding quitting, or prescribing cessation medication. Effectiveness was defined as the number of current smokers at baseline who engaged in tobacco treatment and reported abstinence from smoking for at least 7 days, among responders at 6-month follow-up. Additional details about outcome measures are reported elsewhere [31].

### Estimating resources and costs

The present study focused on operating (maintenance) costs—i.e., costs to maintain the program after it was developed—as they are most relevant to decision makers. Planning/development costs (e.g., EHR modifications) were inconsistently reported by sites and were not included in the analysis. Research-related costs were excluded as they are not relevant to replicating the program in another setting. The following categories of cost measures were reported by centers: program personnel type and effort, medications provided/covered by the program, educational and training materials, software and technology services (e.g., interactive voice response system), equipment (e.g., computers), and office space. As stated previously, participating sites reported costs for at least one 6*-*month reporting period, up to a maximum of 3 reporting periods from 2018 to 2020.

### Cost analysis

We calculated the total operating costs for each site within a 6-month time period by summing reported expenses across all categories. Costs were reported from the perspective of the health system, which is most relevant to decision makers in the case of tobacco treatment program implementation within cancer care settings, as opposed to the patient or societal perspectives. All costs were expressed in local market terms following the guide to costing behavioral interventions developed by Ritzwoller et al. [44]. For the personnel category, effort dedicated to delivering the tobacco treatment program was reported by type of personnel and multiplied by the average wage rates for each personnel type as provided by the Bureau of Labor Statistics [45] for the appropriate metropolitan statistical area of the center. This approach was used to minimize the impact that site-specific variations may have had on intervention cost estimates. Fringe benefits were estimated at 30% of total salary costs for each site. Values were adjusted to 2020 US dollars using the consumer price index to account for inflation [46]. For sites that reported costs for more than one time period, we reported the average cost per category across all available time periods. We converted all costs to monthly costs. We then calculated the cost-per-participant and cost-per-quit for each site by dividing total operating costs by (1) the number of patients that engaged in tobacco treatment program services within a 6-month period, and (2) the number of patients who reported seven-day abstinence from tobacco use over a 6-month time period, respectively. To calculate these two ratios, we used the reach and effectiveness measures reported in the most recent time period, for sites that reported them in more than one time period. Cost-per-quit was unavailable for 3 sites that had not yet reported effectiveness data.

### Site visit interviews

Researchers from the C3I Coordinating Center with expertise in qualitative methodology, tobacco cessation, and health systems research developed the interview guide, informed by the Consolidated Framework for Implementation Research (CFIR) [47]. Copies of the interview guide are available from the Coordinating Center upon request. Program leaders and staff were asked about program components, the implementation process, barriers and facilitators, and the resources needed to maintain the program over time. Interviews followed a semi-structured format in which respondents were asked the same questions with the opportunity for customized follow-up and suggested probes depending on responses. Interviews were transcribed verbatim, cross-checked by another team member, and analyzed by a team of two researchers using NVivo version 11 (QSR International), a qualitative data management program.

### Qualitative data analysis

The research team first conducted queries of coded transcripts from sites that reported cost data to extract content relevant to implementation barriers, implementation facilitators, and resources needed for program sustainability. Queries underwent secondary inductive coding for emerging themes, which were compiled and grouped according to constructs in the Consolidated Framework for Implementation Research (CFIR) [47] related to intervention characteristics, inner setting, and implementation process. Themes most relevant to understanding costs and resource needs for implementation were identified and added to the site-level matrix, allowing for a side-by-side comparison of quantitative and qualitative findings on costs specific to each site. Interpretation of findings followed a concurrent mixed methods approach, whereby qualitative findings complemented quantitative findings with information on contextual factors related to costs of implementation; and (2) intangible resource factors (e.g., time, space) that are difficult to collect in a quantitative format.

## Results

### Tobacco treatment program services

Table 1 presents the tobacco treatment program components offered at each site, numbered 1 through 15. Among the 15 sites, 10 sites were from Cohort 1 (2017–2019) and 5 sites were from Cohort 2 (2018–2020). Tobacco treatment programs at these sites were launched in 2017 or earlier (3 sites), 2018 (8 sites), and 2019 (4 sites, results not shown). The number of treatment components offered varied widely across centers. For example, Site 4 offered only individually delivered in-person tobacco treatment and referral to the quitline, whereas Site 14 offered all treatment components. Among the 15 sites, 13 offered in-person counseling (either individually or group-delivered), and 9 sites offered telephone-based counseling. In addition, 8 sites offered point-of-care interventions, 3 deployed an interactive voice response system, 12 referred patients to the state quitline, 5 offered the SmokefreeTXT service, 7 offered web-based resources, and 14 offered patients cessation medications.

**Table 1:** Tobacco treatment program types offered at each site

Site	Site	In-person counseling	Telephone-based counseling	Point of care	Interactive voice response system	Referral to quitline	Smokefree TXT	Web resource (e.g., smokefree.gov)	Cessation medications
**8**^a^	**1**^a^	✓		✓		✓	✓	✓	✓
**14**^a^	**2**^a^	✓	✓	✓	✓	✓	✓	✓	✓
**15**^b^	**3**^b^	✓				✓			✓
**5**^a^	**4**^a^		✓	✓					✓
**6**^a^	**5**^a^	✓	✓	✓	✓	✓			✓
**12**^b^	**6**^b^	✓	✓			✓	✓	✓	✓
**7**^a^	**7**^a^					✓			✓
**1**^a^	**8**^a^	✓	✓	✓					✓
**11**^b^	**9**^b^	✓	✓	✓		✓	✓	✓	✓
**4**^a^	**10**^a^	✓				✓			
**2**^a^	**11**^a^	✓	✓	✓		✓			✓
**10**^b^	**12**^b^	✓	✓	✓		✓		✓	✓
**13**^b^	**13**^b^	✓				✓		✓	✓
**9**^a^	**14**^a^	✓			✓	✓	✓		✓
**3**^a^	**15**^a^	✓	✓					✓	✓

^a^Cohort 1 site (2017–2019)

^b^Cohort 2 site (2018–2020)

### Costs per site

Monthly operating costs are reported in Table 2. Total operating costs per month ranged from $5129 to $20,751, with a median of $11,045. The highest costs were typically reported in the personnel category, which ranged from $4122 to $19,794 with a median of $10,307 (representing 90% of median total monthly costs). Only 5 sites reported medication costs to the health system, ranging from $17 to $573 in total monthly costs. While some patients may have had coverage for cessation medications through their health plans, select programs reported offering medications as a treatment benefit. Additionally, 10 sites reported educational material costs, ranging from $6 to $435 per month; 12 sites reported training costs, ranging from $96 to $516 per month; 6 sites reported technology costs, ranging from $171 to $4167 per month; 12 sites reported equipment costs, ranging from $10 to $620 per month; and 6 sites reported other operating costs (e.g., biochemical verification), ranging from $36 to $337 per month.

**Table 2:** Monthly operating costs ($) per site, by expense category and (%) of total cost

Site	Personnel	Medications	Materials	Training	Technology	Equipment	Other	Total cost	Reporting periods
1	10,307 (95.1)	–	17 (0.2)	–	513 (4.7)	–	–	10,838	2
2	19,794 (95.4)	–	131 (0.6)	282 (1.4)	171 (0.8)	79 (0.4)	337 (1.6)	20,751	3
3	6878 (62.3)	–	–	–	4167 (37.7)	–	–	11,045	1
4	11,744 (90.0)	–	–	513 (3.9)	257 (2.0)	539 (4.1)	–	13,052	1
5	13,518 (95.5)	–	6 (0.0)	175 (1.2)	–	453 (3.2)	–	14,152	3
6	17,542 (99.0)	–	168 (1.0)	–	–	–	–	17,710	2
7	8400 (90.2)	454 (4.9)	7 (0.1)	134 (1.4)	–	268 (2.9)	50 (0.5)	9314	1
8	6641 (86.9)	–	–	390 (5.1)	–	611 (8.0)	–	7642	1
9	17,455 (92.8)	17 (0.1)	35 (0.2)	485 (2.6)	–	537 (2.9)	288 (1.5)	18,817	3
10	5881 (91.1)	–	–	376 (5.8)	–	196 (3.0)	–	6453	1
11	11,046 (97.2)	–	178 (1.6)	96 (0.8)	–	10 (0.1)	36 (0.3)	11,367	3
12	8346 (82.7)	573 (5.7)	366 (3.7)	377 (3.8)	–	227 (2.3)	122 (1.2)	10,010	3
13	4558 (88.9)	–	–	220 (4.3)	–	186 (3.6)	164 (3.2)	5129	2
14	4122 (48.8)	121 (1.4)	309 (3.7)	516 (6.1)	2759 (32.7)	620 (7.3)	–	8447	2
15	14,103 (87.9)	51 (0.3)	435 (2.7)	359 (2.2)	889 (5.4)	209 (1.3)	–	16,046	1
Median	10,307 (90.0)	0	17 (0.1)	282 (2.2)	0	209 (1.6)	0	11,045	
Minimum	4122	0	0	0	0	10	0	5129	
Maximum	19,794	573	435	516	4167	620	337	20,751	

### Cost per participant and cost per quit

Table 3 presents cost-per-participant and cost-per-quit across sites. The number of participants, varied widely, ranging from 32 to 935 participants per site, with patient engagement ranging from 2.1% to 85%. The quit rate (i.e., effectiveness) also varied by site from a low of 6.7% to a high of 34.4%. Cost-per-participant ranged from $70 to $2093, with a median of $466, while cost-per-quit ranged from $330 to $9628, with a median of $2688. Cost-per-quit was less than $3500 at 9 of the 12 sites that had available data.

**Table 3:** Cost per participant and cost per quit across sites, in descending order by number of treatment participants

Site	Patients, current smokers (*n*)	Treatment participants (*n*)	Patient engagement (%)	Cost per participant ($)	Quits (*n*)^a^	Quit rate (%)	Cost per quit ($)
1	2847	935	32.8	70	197	21.1	330
2	2342	557	23.8	224	57	10.2	2184
3	1591	404	25.4	164	43	10.6	1541
4	702	360	51.3	218	24	6.7	3263
5	2499	247	9.9	344	40	16.2	2123
6	570	198	34.7	537	38	19.2	2796
7^b^	1694	120	7.1	466	–	–	–
8	239	96	40.2	478	19	19.8	2413
9	1268	90	7.1	1254	31	34.4	3642
10	460	86	18.7	450	14	16.3	2766
11	512	75	14.6	909	17	22.7	4012
12	293	74	25.3	812	23	31.1	2611
13^b^	1489	67	4.5	459	–	–	–
14^b^	178	65	36.5	780	–	–	–
15	203	46	22.7	2093	10	21.7	9628
Median	702	96	23.8	466	28	19.2	2688
Minimum	178	46	4.5	70	10	6.7	330
Maximum	2847	935	51.3	2093	197	34.4	9628

^a^The number of participants and number of quits were based on the most recent reporting period. The quit rates were calculated based on 7-day point-prevalence among participants who responded to 6 months follow-up. Quits (*n*) were observed during the same period but represent the patients enrolled in the previous 6 months

^b^Number of quits was not reported for these sites

### Contextual cost-related factors influencing program implementation and delivery

Site interviews provided context for understanding costs related to personnel, technology, and equipment, and complemented quantitative findings with information about other resource-related factors such as available time and space. These factors were identified as barriers and facilitators to program implementation and were also mentioned in response to questions regarding program needs for sustainability. In terms of overall program funding, sites mentioned several facilitators, including initial funding support from the NCI and matching funds from the cancer center or healthcare system (e.g., Sites 7, 12, and 15), as well as industry support in the form of cessation medication donations (e.g., Site 15). Several sites that received an initial commitment of funding support from either the cancer center or healthcare system (e.g., Sites 4, 7, 8, 11, and 14) were also considering business models for sustainability that would include billing for tobacco treatment services. Meanwhile, Site 8 embraced a paradigm shift to point of care smoking cessation as a strategy to address suboptimal referral rates and unsustainable program funding. Site 5, which also received a commitment from the cancer center for additional funding, expressed concern about the pressures of seeking grant funding to sustain the program. Respondents at all sites were asked what resources they would need to sustain their program in the long-term. Responses revealed needed resources, which were not yet available at the site but believed to be necessary for sustainability. Specific staffing needs included the need for additional full-time tobacco treatment specialists to “reach scale” or to administer telephone counseling, a community educator, a “dedicated person for cancer,” support staff after the end of supplement funding, and staff dedicated to long-term follow-up.

## Discussion

This study is the first to our knowledge to examine the economic implications of implementing tobacco treatment programs within cancer centers on a national scale. Health care decision makers often request information showing how a new treatment program will affect their budget. Accordingly, economic evaluations are important to guiding the implementation and sustainment of tobacco treatment programs in oncology and other clinical settings.

Implementing and sustaining tobacco treatment programs in cancer centers requires resources, most notably personnel time, which are often not documented when the results of the program implementation are presented in the literature. In the present mixed methods study, we examined the costs of implementing tobacco treatment programs at 15 NCI-designated cancer centers participating in the C3I, along with the contextual information about the resources and other related costs, such as time and space, associated with these implementation efforts. These programs had a median monthly cost of $11,045, with the bulk of costs dedicated to program personnel. The findings suggest that tobacco treatment programs implemented in cancer care settings achieve noteworthy quit rates for relatively modest costs. While many of the participating centers were in the early stages of implementation, the median cost-per-quit was $2781 among sites with available data. In comparison, the cost-per-quit based on implementing the clinical practice guideline for tobacco treatment was estimated at $3779 (in 1995 dollars), equivalent to $6500 (in 2020 dollars) [37]. Overall, the cost-effectiveness of these programs is expected to improve as programs mature and achieve greater efficiency and higher patient engagement over time.

It is noteworthy that the cost-per-quit at many C3I sites is generally within the range of historical cost-effectiveness estimates for tobacco treatment, even though each of the sites implemented markedly different tobacco treatment program components. More specifically, Site 8 achieved the lowest cost-per-quit using a point-of-care model in which oncology providers referred patients to quitline services. The point-of-care intervention was considered part of standard care and did not contribute costs to the tobacco treatment program. Yet, Site 5 had one of the lowest cost-per-quit rates despite primarily offering individually delivered counseling services, arguably the most labor-intensive option. In comparison, Site 2 achieved modest cost-per-quit while offering all categories of tobacco treatment components, by taking a dual approach of promoting high reach services as a population-based approach while offering high-intensity services to a subgroup of patients. Meanwhile, the remaining sites offered a subset of services that included a combination of more-intensive and less-intensive options. In addition, cost-per-quit across all programs is lower than the incremental costs attributed to failure of first-line cancer treatment associated with smoking after a cancer diagnosi s[4]. As these programs mature over time, an important empirical question is whether there will be more divergence in cost-per-quit according to program components, characteristics of patients served and geographic location of cancer centers. Meanwhile, the higher cost-per-participant and cost-per-quit observed at other sites may be at least in part due to the fact that these programs could still be in the early stages of implementation and may not have reached full capacity. Additionally, quit rates could be underreported across sites, as some tobacco treatment participants who quit may have been lost to follow up at 6 months.

Given budget constraints, sites must balance between prioritizing program reach and effectiveness. Among sites that achieved similar cost-per-quit rates, a subset of sites reported high quit rates (e.g., 34%) while engaging a relatively small number of patients (i.e., fewer than 100 participants). Alternatively, other sites have achieved high engagement (e.g., more than 500 participants) with lower quit rates (e.g., 10%). Given that the C3I did not dictate uniform program components across all sites, centers had the freedom to choose program designs that optimize fit within the local context. For example, several sites achieved low cost-per-quit by relying heavily on referrals to the state quitline. However, variations across state quitlines in terms of funding, services provided, and eligibility criteria suggest that this may not be an equally reliable option for other cancer centers. Overall, several sites achieved above-average rates in both engagement and effectiveness (Sites 1, 6, 8, and 12) while maintaining relatively low cost-per-quit (less than $3000). Whereas the clinical practice guideline for tobacco treatment has been clear about the effectiveness of tobacco cessation interventions, the C3I experience suggests that different intervention forms may achieve similar cost-effectiveness. Future research should further examine the relative cost-effectiveness of different tobacco treatment intervention forms with more granular data that include patient-level metrics for the various tobacco treatment services offered at each site.

Despite convincing evidence on the cost-effectiveness of tobacco treatment interventions [36] and a high willingness to support them by clinicians [48], implementation of tobacco treatment programs into clinical oncology practice might be hampered due to specific economic barriers that represent disincentives for health systems to implement such programs. One key issue in the implementation and dissemination of evidence-based practices in clinical settings is the identification of funding sources for program development and sustainment. Whereas the C3I has provided funding for the development of tobacco treatment programs at cancer centers, the sustainability of these programs remains a challenge. However, as a condition of participation in the C3I, centers made a commitment to maintain their programs beyond the initial period of NCI support. This initial commitment from the centers has been secured largely with institutional funds, however many sites have identified opportunities for partial cost recovery, mainly by seeking reimbursement for tobacco treatment services.

Tobacco treatment is an example of an intervention for which the evidence on the value and cost-effectiveness is convincing [36], and therefore, implementation in clinical practice is warranted. As an example, findings from the current study serve as a reminder that pharmacotherapy—an intervention with demonstrated population-level effectiveness in cancer patients [49] and offered by the majority of the participating sites—can be offered at low or no cost to the health care system. However, the lack of explicit priority-setting about implementation of tobacco treatment and the potential conflicts among the stakeholders in the health system are critical barriers. Also, behavioral factors in individual health professionals, such as clinical inertia and persistent routine behaviors, may inhibit change. Therefore, implementation of tobacco treatment programs as evidence-based, cost-effective practices does not follow automatically, as there are barriers for change at multiple levels that must be addressed [50–52].

Additional research is needed to improve the understanding of economic barriers and facilitators to the adoption and sustainment of tobacco treatment programs in oncology settings. Future research should also consider strategies to inform decision makers on coverage for tobacco treatment as an alternative for current fee-for-service models that do not allow for the integration of sustainable intensive tobacco cessation counseling into cancer care [53]. Given that fee-for-service reimbursement is predicated upon evaluation and management physician billing codes for treatment, intensive counseling delivered by unlicensed tobacco treatment specialists can only be reimbursed at low rates using preventive counseling codes. The current reimbursement rates do not incentivize additional counseling and are insufficient to sustain intensive cessation counseling [53]. However, programs that employ tobacco treatment specialists with either a social work degree (e.g., LCSW) or psychology degree (e.g., PhD/PsyD) can indeed bill at higher rates, but face higher labor costs. Accordingly, economic methods can play a critical role to support the business case for sustainable tobacco treatment programs as a worthwhile investment of the limited resources of health systems. Further, the recent diffusion of digital and telehealth approaches offers opportunities to enhance the cost-effectiveness of tobacco treatment over more in-person, high-touch approaches.

The present study had a number of strengths, including the evaluation of tobacco treatment program implementation costs within a large cohort of cancer centers across the US, comprehensive cost estimates for these programs, and the use of quantitative and qualitative interview data to estimate and interpret the comparative cost-effectiveness of tobacco treatment programs. The C3I is the first initiative of its kind and has been leading the implementation of evidence-based tobacco treatment programs across NCI-designated cancer centers nationwide. The initiative has produced unique information that can improve our understanding of tobacco treatment in oncology settings, including implementation costs. The mixed methods approach used in this study provides insights on contextual information and stakeholder perspectives beyond findings captured by monetary values alone.

Limitations included the retrospective nature of cost data collection. The level of accuracy of reported costs may have been compromised due to retrospective cost reporting and the fact that tobacco treatment resources may have been shared by oncology and other units within the health system. Whereas most sites focused their C3I efforts on the outpatient setting, where it is easier to track cancer patients separately from other patients, some C3I sites also included tobacco treatment programs in the inpatient setting, where tobacco treatment resources may be shared with non-cancer patients. In those instances, it was more challenging to isolate the resources that were attributable to treating tobacco use specifically among cancer patients, which may have led to overestimating cost-per-quit. Similarly, at least one cancer center (Site 12) provides tobacco treatment to other outpatient specialties, incurring costs without capturing those patients’ outcomes data in C3I reporting. Among the many centers that referred patients to the quitline, cost-per-quit may depend on the quality of the quitline available in their state. As the present analysis was limited to the health system perspective, it does not reflect the actual public cost of the tobacco treatment programs. In addition, program effectiveness is measured by short-term assessment of quits, which may differ over the long term. As such, long-term assessment of both costs and outcomes is required to fully explore the cost-effectiveness of the tobacco treatments offered. Higher intensity interventions may be more costly to implement, but they may also be more effective over time.

Although all sites reported the range of tobacco treatment services they offered, the reporting was not granular enough to reflect specific treatment enrollment at the patient level. Therefore, we were unable to ascertain the relative effectiveness and cost-effectiveness of treatment services at the site level. Also, given the variations across programs, we were unable to estimate the marginal impact of each component. Further, given that cost reporting was voluntary for C3I sites, response bias may be a potential factor. Although sites may not have participated in cost reporting because they had not sufficiently progressed in implementation at the time of this report, confounding factors (e.g., limited personnel) may have contributed to other sites not reporting their cost information. This limitation may have been exacerbated by the COVID-19 pandemic in the 2020 reporting period. In addition, the C3I is an ongoing initiative, and effectiveness data were unavailable for some sites. Also, with respect to effectiveness, quit rates were based on patient self-report and abstinence was not biochemically verified at the vast majority of sites. Therefore, the accuracy of quit rates may be questionable. Finally, findings from this study were based on the experiences of NCI-designated cancer centers, which typically have more resources than other cancer centers. Although study findings may not be entirely generalizable to other cancer centers, the implementation costs as well as the resource-related barriers and facilitators summarized in this study could inform the implementation of tobacco treatment programs across all cancer centers.

## Conclusions

Costs are a key consideration in the decision to offer tobacco treatment services within health care delivery settings, and oncology care is no exception. Overall, tobacco treatment programs implemented within NCI-designated cancer centers resulted in modest cost-per-quit, regardless of program design. These findings can inform and guide program developers, providers, and implementers with the design and implementation of these programs in similar target populations while ensuring resources are efficiently allocated to maximize value in cancer care. The demonstrable value of tobacco treatment programs should serve to inform the system-level change that is needed to support the sustainment of smoking cessation services in cancer care.

## Data Availability

The datasets generated and analyzed during the study are not publicly available due to the sensitive nature of cost data but are available from the C3I coordinating center on reasonable request.

## Abbreviations

C3I: Cancer center cessation initiative
NCI: National cancer institute
CHEERS: Consolidated health economic evaluation reporting standards
EHR: Electronic health record
